# The leukemia associated nuclear corepressor ETO homologue genes *MTG16 *and *MTGR1 *are regulated differently in hematopoietic cells

**DOI:** 10.1186/1471-2199-13-11

**Published:** 2012-03-23

**Authors:** Ram Ajore, Parveen Kumar, Rakesh Singh Dhanda, Urban Gullberg, Inge Olsson

**Affiliations:** 1 Department of Hematology, C14, BMC, S-221 84, Lund, Sweden; 2 Department of Translational and Regenerative Medicine, Postgraduate Institute of Medical Education and Research, Chandigarh 160 012, India

## Abstract

**Background:**

MTG16, MTGR1 and ETO are nuclear transcriptional corepressors of the human ETO protein family. MTG16 is implicated in hematopoietic development and in controlling erythropoiesis/megakaryopoiesis. Furthermore, ETO homologue genes are 3'participants in leukemia fusions generated by chromosomal translocations responsible of hematopoietic dysregulation. We tried to identify structural and functional promoter elements of *MTG16 *and *MTGR1 *genes in order to find associations between their regulation and hematopoiesis.

**Results:**

5' deletion examinations and luciferase reporter gene studies indicated that a 492 bp sequence upstream of the transcription start site is essential for transcriptional activity by the *MTG16 *promoter. The TATA- and CCAAT-less promoter with a GC box close to the start site showed strong reporter activity when examined in erythroid/megakaryocytic cells. Mutation of an evolutionary conserved GATA -301 consensus binding site repressed promoter function. Furthermore, results from *in vitro *antibody-enhanced electrophoretic mobility shift assay and *in vivo *chromatin immunoprecipitation indicated binding of GATA-1 to the GATA -301 site. A role of GATA-1 was also supported by transfection of small interfering RNA, which diminished *MTG16 *expression. Furthermore, expression of the transcription factor HERP2, which represses GATA-1, produced strong inhibition of the *MTG16 *promoter reporter consistent with a role of GATA-1 in transcriptional activation. The TATA-less and CCAAT-less *MTGR1 *promoter retained most of the transcriptional activity within a -308 to -207 bp region with a GC-box-rich sequence containing multiple SP1 binding sites reminiscent of a housekeeping gene with constitutive expression. However, mutations of individual SP1 binding sites did not repress promoter function; multiple active SP1 binding sites may be required to safeguard constitutive *MTGR1 *transcriptional activity. The observed repression of *MTG16*/*MTGR1 *promoters by the leukemia associated *AML1*-*ETO *fusion gene may have a role in hematopoietic dysfunction of leukemia.

**Conclusions:**

An evolutionary conserved GATA binding site is critical in transcriptional regulation of the *MTG16 *promoter. In contrast, the *MTGR1 *gene depends on a GC-box-rich sequence for transcriptional regulation and possible ubiquitous expression. Our results demonstrate that the *ETO *homologue promoters are regulated differently consistent with hematopoietic cell-type- specific expression and function.

## Background

The highly conserved *ETO *(Eight-Twenty-One) corepressor gene family traced to Drosophila ETO homologue *Nervy *[1] contains the myeloid translocation gene (MTG) 16 *(MTG16) (*gene name *CBFA2T3*) or murine *ETO-2 *(gene name *cbfa2t3*), *MTG8 (*gene name *CBFA2T1*, *RUNXT1, ETO*) and MTG-related protein 1 (*MTGR1*) *(*gene name *CBFA2T2)*. The ETO homologues do not bind directly to DNA but rather function as protein scaffolds and bring about gene repression indirectly through an interplay with multiple transcriptional regulatory proteins [2,3]. MTG16 has been shown to interact with canonical transcription factors such as PLZF, BCL6, TAL1/SCL, Gfi1and Heb [4-9], and MTGR1 with TAL1/SCL [10]. The ETO homologues also recruite chromatin regulating proteins such as nuclear corepressors [3,11,12] and histone deacetylases (HDACs) that catalyze chromatin modifications, resulting in transcriptional repression. Importantly, *ETO *homologue genes are involved in chromosomal translocations of acute leukemia, *MTG16 *in the generation of the *AML1- MTG16 *fusion gene of t(16;21) [13] in patients with therapy-induced leukemia, *ETO *in the generation of the *AML1-ETO *fusion gene of t(8;21) [14,15] and *MTGR1 *in the generation of *AML1*-*MTGR1 *fusion gene of t(20;21) [16]. The leukemic fusion proteins so encoded disrupt normal function of transcriptional regulators, to promote dysregulation of hematopoietic cell differentiation [17].

Expression of the MTG16 (murine ETO-2) corepressor is confined to early hematopoiesis whereas MTGR1 is present throughout hematopoietic maturation [18]. Thus, ETO-2 increases during differentiation of murine embryonic stem cells into hematopoietic cells [19] suggesting a role in the development of the blood cell compartment. This is consistent with *MTG16 *being the most highly expressed *ETO *homologue gene in the stem/progenitor cell compartment [18]. Furthermore, MTG16/ETO-2 has a role in controlling erythropoiesis and megakaryopoiesis. In this context, MTG16/ETO-2 is incorporated in Ldb1 (LIM domain- binding protein 1) and TAL1 (T-cell acute lymphocytic leukemia protein 1) containing transcription factor complexes [5,8,20,21], mediating transcriptional suppression. As a heteromer with MTG16, also MTGR1 complexes with TAL1 in erythroid cells, enhancing transcriptional repression [10]. The ETO-2 recruitment within nuclear complexes may determine the onset of terminal erythroid/megakaryocytic differentiation [5], while the downmodulation of *MTG16/ETO-2 *at late stages of erythropoiesis suggests that decreased transcriptional repression is necessary for late, terminal erythroid differentiation to occur. For example, in erythroid MEL cells, induction of differentiation is accompanied by dissociation of nuclear protein complexes concomitant with a decrease in the relative level of ETO-2 [8]. Moreover, shedding the negative regulator ETO-2 [21] transforms the Ldb complex into a positive regulator of final erythroid differentiation, supporting the notion that multi-protein complexes may keep erythroid target genes suppressed until corepressor ETO-2 is eliminated at final differentiation [21]. Similarly, in megakaryopoiesis the ETO-2 expression is restricted to immature cells consonant with a function in repression of genes that should be only terminally expressed [22]. Finally, a physiological hematopoietic role for MTG16 has been proven by results from targeted disruption of murine *MTG16 (ETO-2) *[23]. Although no complete block in hematopoietic differentiation was discovered by *ETO-2 *knockout, a requirement was demonstrated for ETO-2 in fate decisions, proliferation and stress- dependent hematopoiesis. As a corroboration of functional differences between ETO homologues, targeted disruption of MTGR1 showed no phenotypic consequences for hematopoiesis [24].

Endogenous cell-type-specific hematopoietic expression of the ETO homologues [18] suggests differences in gene regulation. The *ETO *promoter is regulated by the GATA transcription factor in erythroid/megakaryocytic cells [25]. We now examined functional promoter elements upstream of the coding sequences for *MTG16 *and *MTGR1*. A critical role in transcriptional regulation was shown for an evolutionary conserved GATA binding site of the *MTG16 *promoter. In contrast, *MTGR1 *has a strong TATA-less promoter with multiple GC box-rich regions of possible importance for constitutive expression. Our results show that the ETO homologue promoters are regulated differently, consistent with cell-type- specific expression.

## Results

### Identification of transcriptional start site and sequence analysis of proximal promoters of *MTG16 *and *MTGR1*

**Figure 1:**
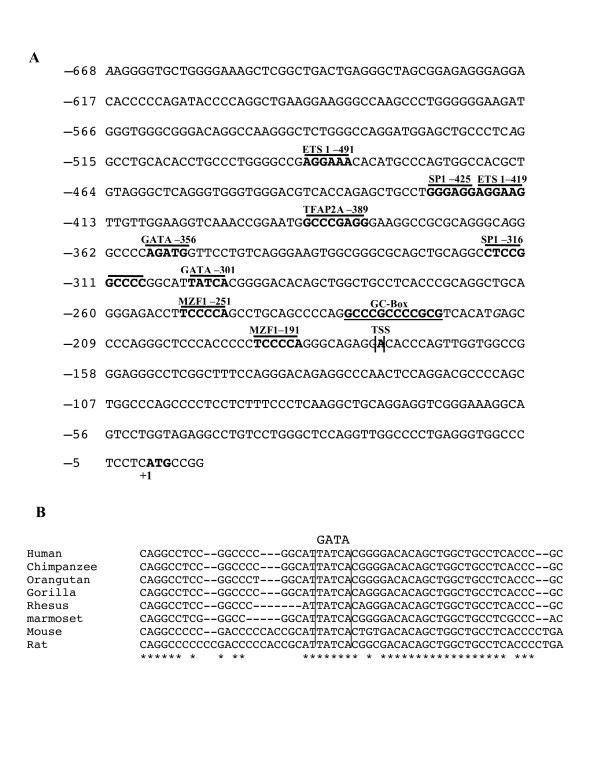
**Localization and structure of *MTG16 *promoter**. (**A**) *Nucleotide sequence of the MTG16 promoter*. Identification of transcription start site (TSS) and amplification of the promoter was performed as described in Methods. Nucleotide +1 is the translational start site (ATG). Putative consensus binding sites for transcription factors identified with MatInspector http://www.genomatix.de//matinspector.html and http://jaspar.genereg.net/ are marked. The promoter lacks TATA and CCAAT boxes, but a GC box (underlined) close to the TSS may be important for start site utilization. (**B**) Homology search was performed for a stretch of sequence from -251 to -499 human sequencewith NCBI nucleotide blast (Blastn) programme in database for "whole-genome shotgun reads" (wgs) and an evolutionary conserved GATA site was identified (marked between straight lines). The following accession numbers appeared containing homologus sequences, Chimpanzee, b| AACZ03106631.1|; Orangutan gb| ABGA01087101.1|; Gorilla, emb| CABD02143420.1|; Rhesus Monkey, gb|AANU01214553.1|; Marmoset, gb|ACFV01163109.1|; Mouse, ref|NT_078575.6|Mm8_78640_37; Rat, ref|NW_047536.2|Rn19_WGA2058_4. The homology sequences were also found at http://genome.ucsc.edu/cgi-bin/hgBlat.

The MTG16 corepressor is associated with developing hematopoiesis and control of erythropoiesis/megakaryopoiesis. However, transcriptional factors regulating the expression of the *MTG16 *and *MTGR1 *genes have not been identified. Therefore, we tried to discover the important *cis*-acting regions and *trans*-acting factors regulating expression in hematopoietic cells. First, to find the position of the transcriptional start site for MTG16, we performed 5'- rapid amplification of cDNA-ends (RACE). MTG16-cDNA amplified from erythropoietic HEL cell mRNA showed a complete sequence match with a region of *MTG16a *transcript (NCBI Ref. Seq: NT_010542.15). The transcription start site (TSS) was determined at -176 bp upstream of the translation start codon ATG at +1 (Figure 1A). The translation initiation in *MTG16b *is located further downstream to that of *MTG16a *as determined by alignment of mRNA sequence of MTG16a (NM_005187.5) and MTG16b (NM_175931.2). Inspection of the proximal promoter upstream of the transcription start site start (-176 bp) revealed several consensus binding elements (Figure 1A) such as potential ETS1 binding sites at -491 and at -419; SP1 binding sites at -425 and at -316; GATA binding sites at -356 and at -301; MZF1 sites at -251 and at -191; TFAP2A at -389 (Figure 1A). No canonical TATA or CCAAT boxes were detected in the region near the transcription start.

However, a conserved GC box close to the transcriptional start site (Figure 1A) may regulate transcriptional initiation. Sequence comparisons showed the GATA binding site at -301 to be conserved between eight species (Figure 1B).

**Figure 2:**
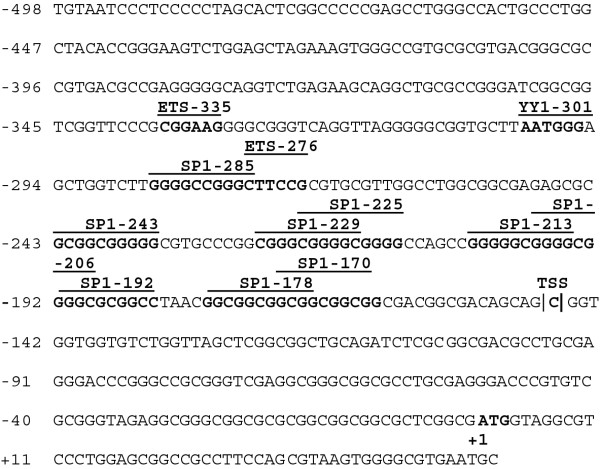
**Localization and structure of *MTGR1 *promoter**. *Nucleotide sequence of the MTGR1 promoter*. The promoter of *MTGR1 *was amplified by PCR as described in Methods. Nucleotide +1 is the translational start site (ATG) and transcription start site at -176 is marked (TSS). The *MTGR1 *promoter lacks TATA and CCAAT boxes but is GC-rich with multiple SP1 binding sites. Putative consensus binding sites for transcription factors identified with MatInspector http://www.genomatix.de//matinspector.html and with http://jaspar.genereg.net/ are marked.

The transcription start site for the *MTGR1 *gene (NCBI Ref. Seq: NM_001032999.2) is present at -146 bp upstream of the translation start codon [16]. As for MTG16, the proximal promoter of MTGR1 lacks canonical TATA and CCAAT boxes. However, the region between -285 to -170 bp contains multiple GC motifs showing strong homology with the classical GC box, 5GGGGCGGGG3' (Figure 2). These boxes are predicted to compose multiple binding sites for SP1. In addition, ETS1 sites are noted at -335 and -276 as well as a YY1 potential binding sites at -301 bp. The different composition of the proximal promoters of *MTG16 *and *MTGR1 *suggests that they are under distinct regulatory mechanisms. While the promoters of both genes lack classical TATA or CCAAT boxes, the presence in the *MTGR1 *promoter of multiple SP1 sites and a CpG island from -450 to -1 bp covering the transcriptional start site (data not shown) indicate characteristics of a housekeeping gene.

### Functional evaluation of MTG16 promoter

**Figure 3:**
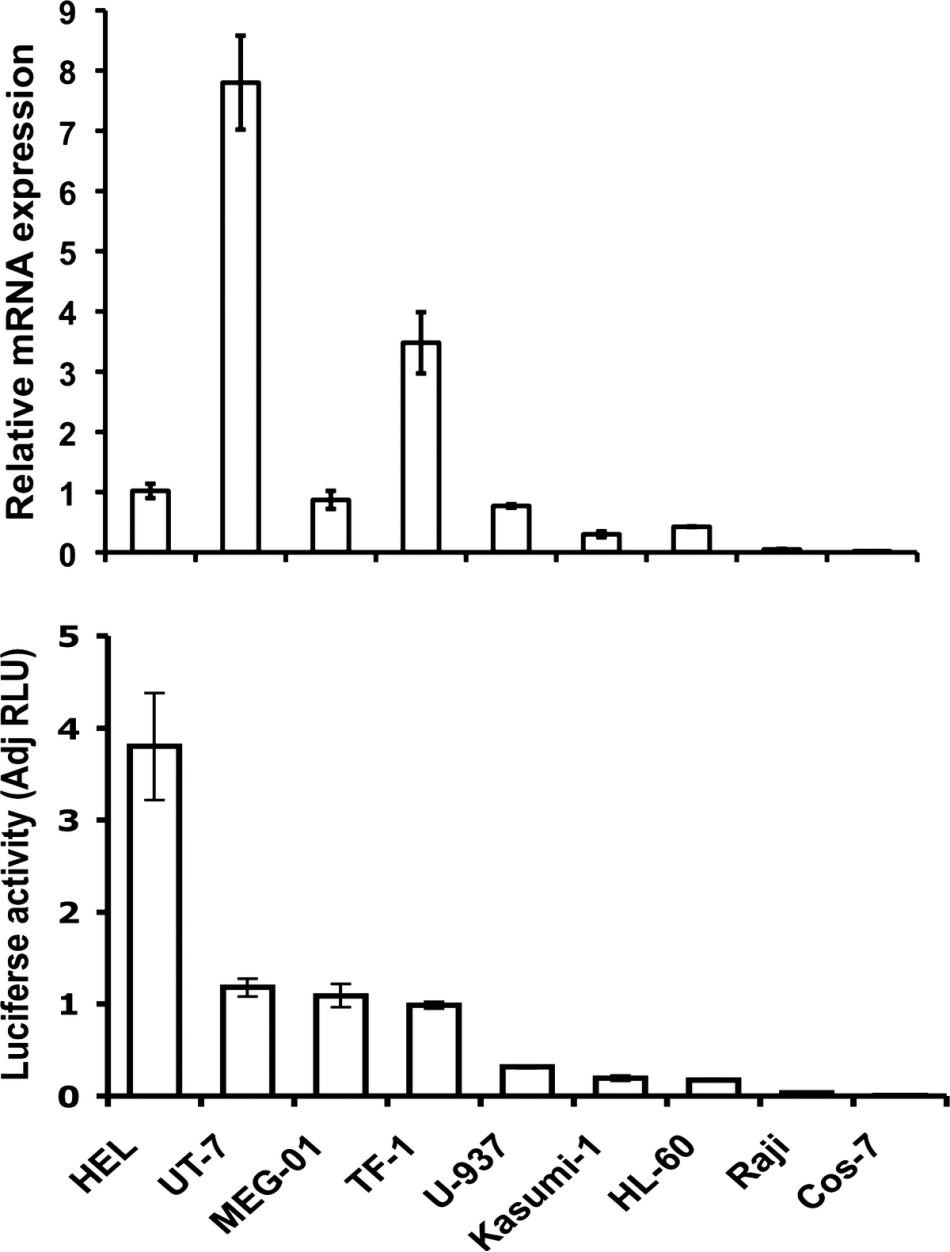
**Reporter gene analysis of the *MTG16 *promoter**. *Promoter activity in different cell lines*. pGL3 -820-57 (-820) was transfected to indicated cell lines and luciferase activity was determined as described in Methods. The relative *MTG16 *mRNA expression of the cell lines is also given. Kasumi, HL-60, Raji and COS cells show both the lowest reporter signals and the lowest *MTG16 *mRNAs.

We cloned a -820 to -57 bp region (translational start codon at +1) of the sequence upstream of the transcription start of the *MTG16 *for determination of transcriptional activity. The cloned region was inserted upstream of the luciferase reporter gene in promoterless pGL3/Basic vector (Promega) to give the pGL3/-820 to -57 plasmid, which was transfected into hematopoietic cell lines and luciferase activity was determined and normalized as described in Methods. pGL3/-820 to -57 showed an approximately 4-fold increased reporter signal as compared to pGL3/SV40-promoter in erythroid HEL cells (Figure 3) indicating strong transcriptional activity in cells expressing the endogenous *MTG16 *gene. The *MTG16 *luciferase reporter also showed a strong signal in other erythroid and in megakaryocytic cell lines investigated (Figure 3). However, no direct correlation was observed between luciferase activity and *MTG16 *transcripts in erythroid and megakaryocytic cell lines. Differences in cellular environment and regulatory elements outside the cloned promoter that modulate endogenous gene expression could explain the lack of direct correlation between promoter activity and gene expression. Promyelocytic HL-60 and acute myeloid leukemia Kasumi-1 cells showed only low luciferase activity; lymphoblastoid Raji cells and monkey kidney COS cells lacked reporter activity. The latter results correlate with low *MTG16 *transcript levels.

### A GATA consensus binding site is critical for *MTG16 *promoter function in erythroid/megakaryocytic cells

**Figure 4:**
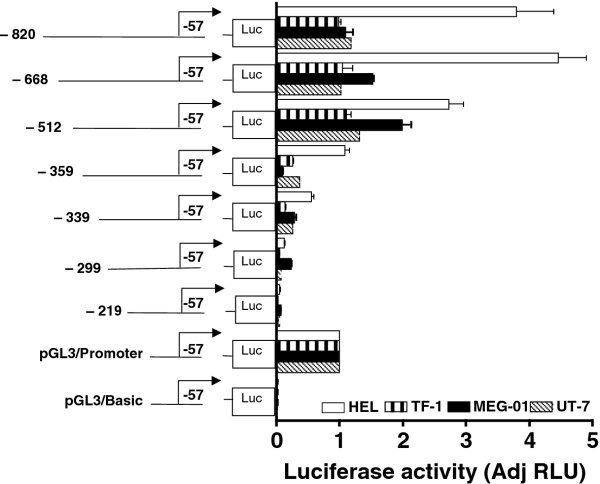
**Functional analysis of the *MTG16 *promoter by sequential 5'-deletion**. The following 5'deleted reporter constructs were generated from the pGL3 -820-57 (-820) construct and examined after expression in erythroid HEL/TF-1, erythroid/megakaryocytic UT-7 and megakaryocytic MEG-01cells: pGL3 -668-57 (-668), pGL3 -512-57 (-512), pGL3 -359-57 (-359), pGL3 -339-57 (-339), pGL3 -299-57 (-299) and pGL3 -219-57 (-219). Promoterless pGL3/basic and the pGL3/SV40-promoter are used as negative and positive control, respectively. The luciferase activity was normalized against pGL3/SV40- promoter activity. Results are shown for 3 to 5 separate transfections; bars represent the mean and the error bars show SEM.

In order to identify important *cis *regulatory elements, a series of constructs was produced by sequential deletions from the 5' end of the -820 to -57 bp region shown in Figure 4. The deletion constructs were incorporated upstream of the luciferase reporter gene in promoterless pGL3/Basic resulting in the pGL3 -820-57, pGL3 -668-57, pGL3 -512-57, pGL3 -359-57, pGL3 -339-57, pGL3 -299-57, and pGL3 -219-57 reporter constructs, which were transfected into erythroid HEL/TF-1, erythroid/megakaryocytic UT-7 and megakaryocytic MEG-01 cells. Deletion of the region from -820 to -668 did not reduce the transcriptional activity of the promoter, while extended 5'-deletions caused a prominent decrease in luciferase activity (Figure 4). We conclude that the region between -668 and -57 bp is likely to contain the major regulatory *cis-*elements of the proximal *MTG16 *promoter, as it was the smallest fragment created with retention of full transcriptional activity (Figure 4).

**Figure 5:**
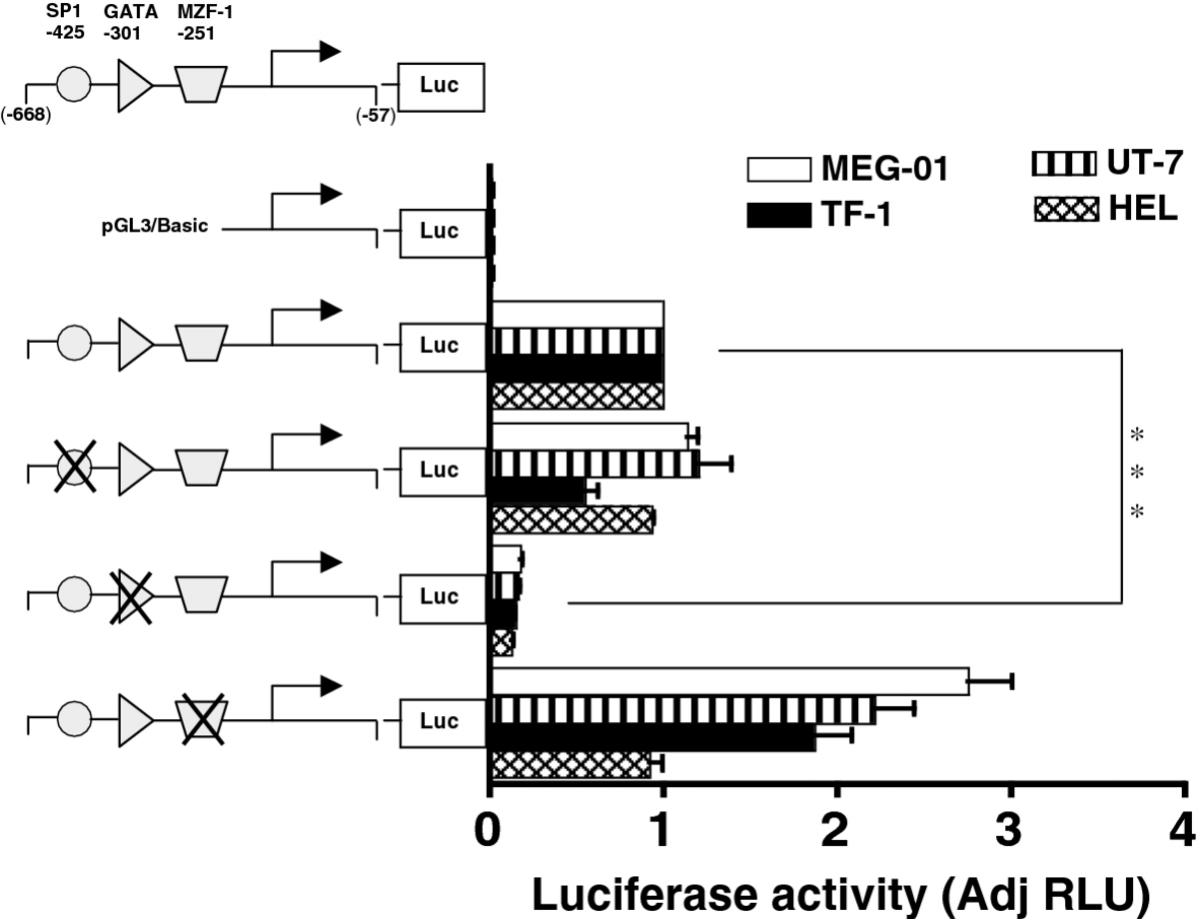
**Functional analysis of putative transcription factor binding sites in the *MTG16 *promoter**. Putative Sp1 (-425), GATA (-301) and MZF-1 (-251) sites in the -668-57 (-668) promoter were individually destroyed by site directed mutagenesis as decribed in Methods. Erythroid HEL/TF-1, erythroid/megakaryocytic UT-7 and megakaryocytic MEG-01cells were transfected and luciferase activity was determined. The pGL3/basic and pGL3/SV40-promoter are used as negative and positive control, respectively. The luciferase activity of mutated *MTG16 *promoter is normalized against luciferase activity of wildtype *MTG16 *promoter.

As noted in Figure 1A potential ETS1, SP1, GATA and MZF1 binding sites are detected in the proximal *MTG16 *promoter. We examined whether these binding sites contribute to promoter transactivation using site-directed mutagenesis. Most notably, disruption of the GATA-301 site led to a 4 to 10-fold reduction in luciferase reporter gene activity in either HEL, TF1, UT-7 or MEG-01 cells relative to intact MTG16 promoter (P < 0.0001) (Figure 5), indicating this GATA site as an important cis-factor for MTG16 gene expression. Furthermore, the GATA -301 site is highly conserved among species (Figure 1B) supporting its functional importance. Moreover, GATA factors are known to play an important role in erythroid/megakaryocytic differentiation [26-28]. Our results suggest that the GATA -301 site may be involved in transcriptional activation in erythroid/megakaryocytic cells.

The GATA-301 site is necessary but not sufficient for transactivation of the *MTG16 *promoter as some deletion constructs (-359, -339) that retain the GATA -301 site do not retain transcriptional activity (Figure 4). However, all mutation experiments were done with the longer -668 construct and its promoter activity was blocked completely by mutation of the GATA-301 site. An element upstream of the GATA-301 site that cooperates with GATA bound to -301 might explain our results. However, no such element was identified as disruption of all upstream potential binding sites that were identified e. g. the SP1 -425, ETS1 -491, ETS1 -419, SP1 -316, and GATA -356 sites did not affect the reporter signal (data not shown). Disruption of the MZF1 -191 sites downstream of the GATA-301 site did not affect the luciferase signal either (data not shown). Mutation of the downstream MZF1 - 251 increased the signal except in HEL cells (data not shown), suggesting that this site possibly mediates binding to some transcriptional repressor.

### GATA-1 binds *in vitro*/*in vivo *to the -301 consensus site in the *MTG16 *promoter

**Figure 6:**
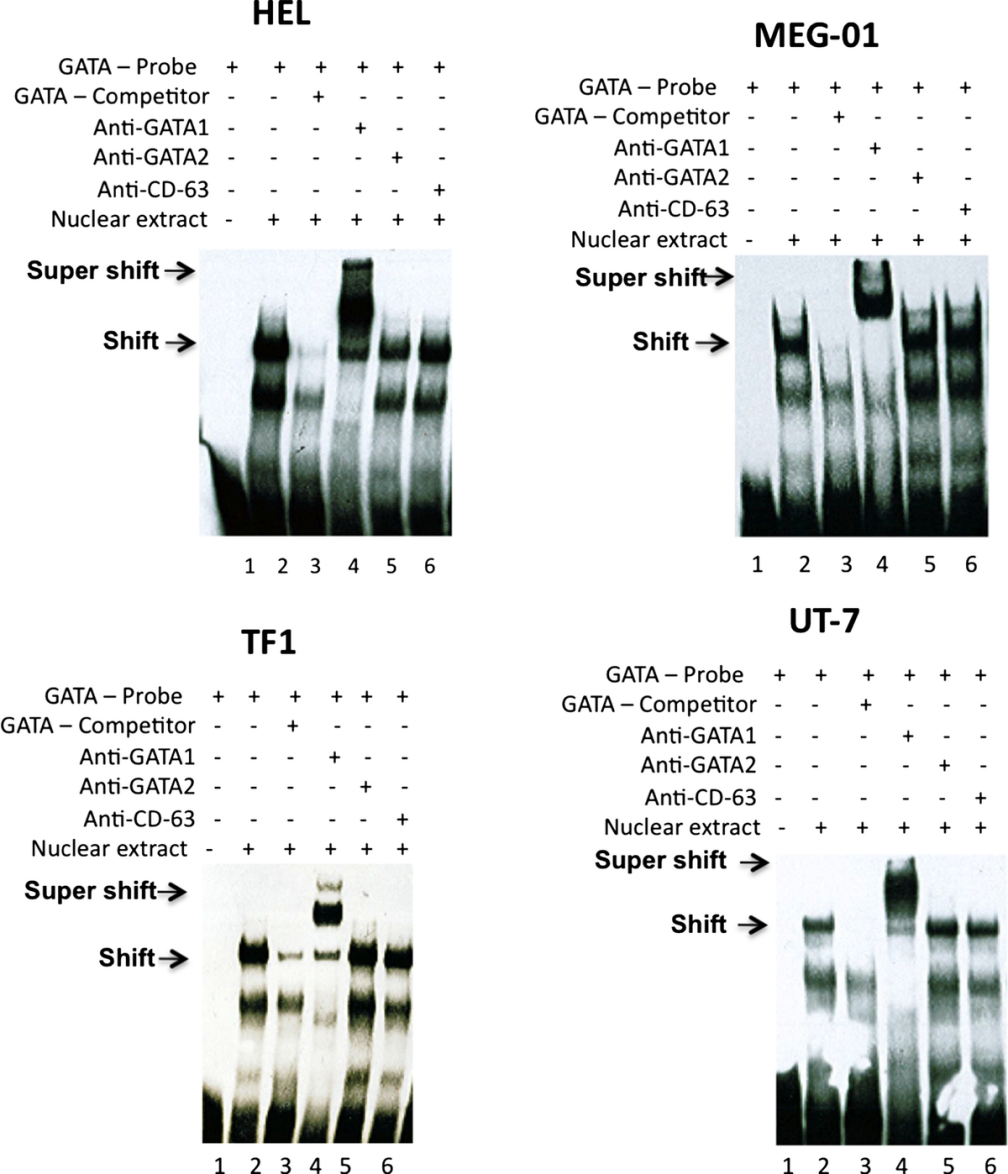
**Detection of DNA-protein interactions using electrophoretic mobility shift/supershift assays *in vitro *of consensus binding sequences in the 5'promoter of *MTG16 *and nuclear extracts**. The following sequence of the oligonucleotide probe was used in EMSA for the conserved GATA (-301) core consensus site (red): **GATA-301 probe 5'- CCCGGCATTATCACGGGGACAC**. Results are given for HEL/MEG-01/TF-1/UT-7 cells. Primary DNA-nuclear protein interactions are shown by arrows marked shift; DNA- nuclear protein-antibody interactions are shown by arrows marked supershift. All cell lines tested showed identical. A shift is shown for the GATA -301 probe that is competed for by excess unlabelled probe (competitor) (3) indicating binding of nuclear extract protein to the biotinylated probe that containing the the GATA -301 sequence. Proteins bound to the probe were "super-shifted" by antibody to GATA-1 (4) but not with antibody to GATA-2 (5). All experiments were repeated at least twice.

The finding that destruction of the conserved GATA (-301) site reduced gene promoter activity (Figure 5) suggested binding of trans-acting factors. Therefore, Electrophoretic Mobility Shift Assays (EMSA) and antibody supershift assays were performed to examine whether the -301 site binds GATA protein *in vitro*. A double-stranded biotin-labeled oligonucleotide probe (see Methods) was prepared for the core consensus GATA -301 site, the sequence and location of which is shown in Figure 6. The oligonucleotide probe was incubated with nuclear extracts prepared from HEL, TF-1, UT-7 or MEG-01 cells to examine interactions between probe and proteins *in vitro*. Nuclear extract proteins from all cell types showed a shift, indicating binding of protein to the biotinylated probe (Figure 6). Binding was specific, as shown by absence of shift in the presence of excess unlabeled competitor probe. Protein bound to the GATA-301 probe was "super-shifted" by anti-GATA-1 antibody, but not with control anti-CD63 antibody (Figure 6). Thus, our results demonstrate that GATA-1 can bind to the consensus GATA-binding site (-301) within the MTG16 promoter. No supershift was observed by anti-GATA-2 antibodies (Figure 6) despite testing of several different antibodies (data not shown), suggesting lack of strong binding of GATA-2 to the consensus site. This finding is similar to previous results from the characterization of the ETO promoter, in which a GATA-site binds GATA-1 but not GATA-2 [25].

In addition to the specific shift, another band is clearly visible in the EMSA (Figure 6). This band-shift is unspecific since it is unaffected or only weakly affected by the competitor probe. However, it obviously contains GATA-1 protein as it is lost by supershifting with anti GATA-1 (Figure 6).

The SP1 -425 and the MZF1 -251 binding sites of the MTG16 promoter were also examined by EMSA. No supershifting was observed by appropriate antibodies suggesting lack of binding of these transcription factors to the respective consensus binding sites (data not shown).

**Figure 7:**
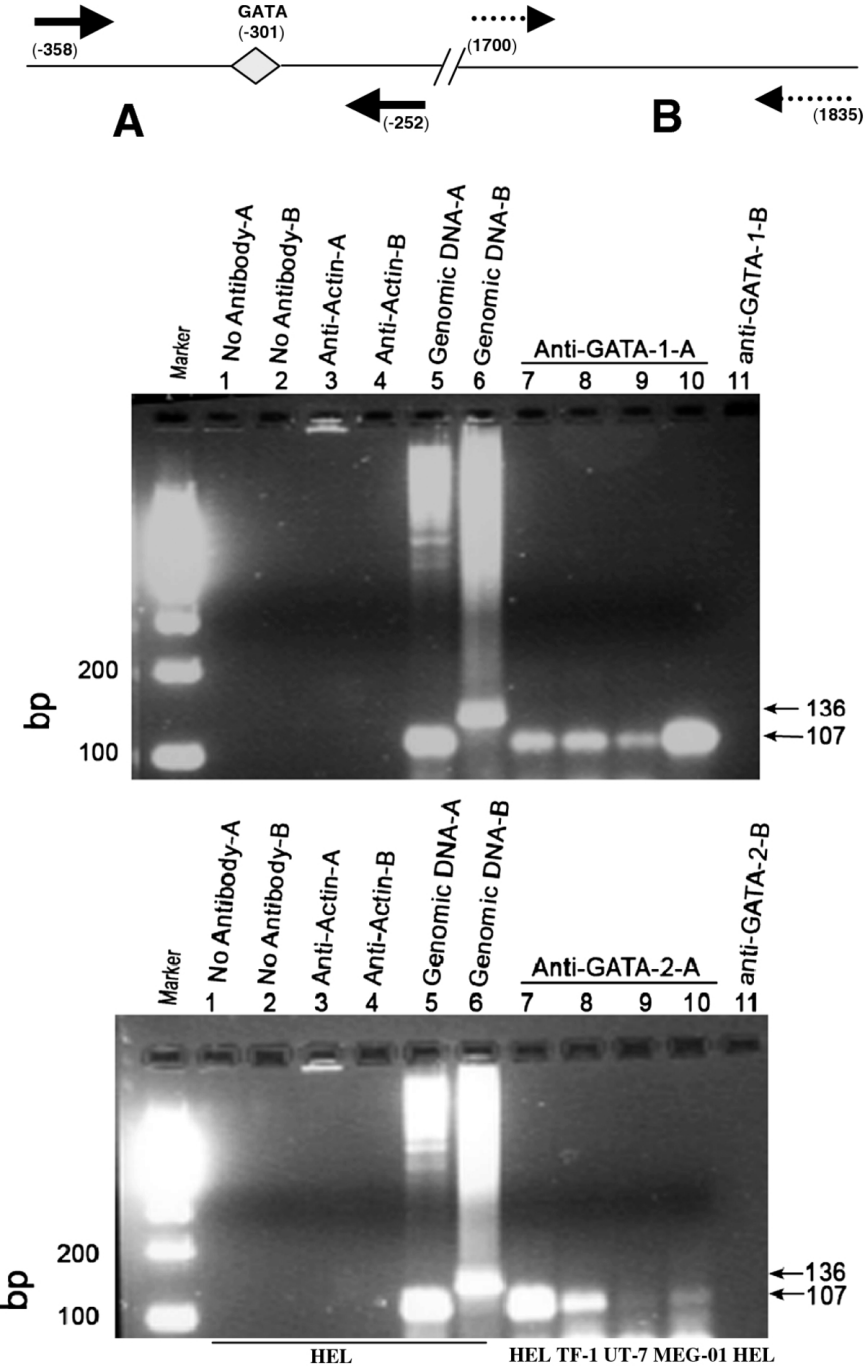
**Chromatin immunoprecipitation (ChIP) assay for examining interactions in vivo of consensus binding sequences in the 5'promoter of *MTG16***. The forward and reverse primers used to amplify the proximal promoter region from -358 to -252 (primers **A**, solid arrows) and forward and reverse primers for a downstream region from 1700 to 1835 as control (primers **B**, dashed arrows) are shown. ChIP assays were carried out as described in Methods using chromatin isolated from erythroid HEL/TF-1, erythroid/megakaryocytic UT-7 and megakaryocytic MEG-01cells. PCR products were separated on a 2% gel and representative results are shown. Lane 1-2, no antibody and primers A or B; lanes 3-4, actin antibody and primers A or B; lanes 5-6, genomic DNA and primers A or B; lanes 7-11, GATA-1(top) or GATA-2 (bottom) antibody and primers A or B (lane 11). By use of the specific primers, a PCR product is generated both from the anti-GATA-1 and the anti- GATA-2 immunoprecipitated chromatin. No amplification is seen without antibody or in the presence of anti-actin. All experiments were repeated at least twice.

Chromatin immunoprecipitation (ChIP) was used to determine whether GATA-1-2 bound to the putative *MTG16 *gene promoter *in vivo*. The assays were carried out with chromatin isolated from HEL, TF-1, UT-7 or MEG-01 cells and antibody towards chromatin-bound GATA-1 or GATA-2. Precipitated DNA was identified by PCR amplification of the MTG16 promoter fragment with gene specific oligonucleotides. By the use of primers specific for the -358 to -252 region, which flank the conserved GATA (-301) site, a 107 bp PCR product was generated using precipitation with either anti-GATA-1 or anti-GATA-2 (but not with anti-actin antibody or with no antibody, serving as negative controls) of all the cell lines examined (Figure 7). Control precipitation with anti-GATA-1 or anti-GATA-2 and amplification of a downstream control region was negative. Thus, specific amplification was achieved after precipitation with both anti-GATA-1 or anti-GATA-2.

Taken together, the results from EMSA/supershift assays demonstrate GATA-1 binding *in vitro *to the GATA -301 binding site and results from ChIP assays demonstrate binding in vivo of both GATA-1 and GATA-2 to the putative *MTG16 *promoter. This result further supports a functional role at least for GATA-1 in activation of the MTG16 promoter, consistent with the results of the mutagenesis studies shown in Figure 5.

### Overexpression of GATA-1

**Figure 8:**
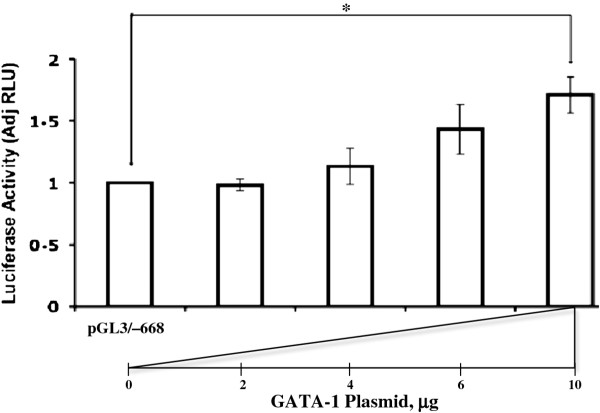
**Effects on the *MTG16 *promoter activity of GATA-1 overexpression**. MEG-01 cells were co-transfected with 15 μg MTG16 -668 to -57 bp promoter plasmid and increasing amounts of GATA-1 plasmid. The luciferase activity is normalized against Renilla and the activity of the MTG16 -668 to -57 bp promoter. The MTG16 promoter is activated by GATA-1 overexpression. The data are from 3 separate experiments. Bars represent the mean and the error bars show SEM. Statistically significant differences are marked by star.

GATA-1 was transiently overexpressed to examine its effect on co-transfected *MTG16 *promoter activity in erythroid/megakaryocytic cells. The reporter signal was increased by overexpression in megakaryocytic MEG-01 cells (p < 0.05) (Figure 8). The specificity of this result is supported by lack of impact by GATA-1 overexpression on the Renilla control (data not shown).

### Repression of *GATA-1 *by siRNA and inhibition of GATA-1 transcriptional activity with HERP2 leads to decreased *MTG16 *promoter activity

**Figure 9:**
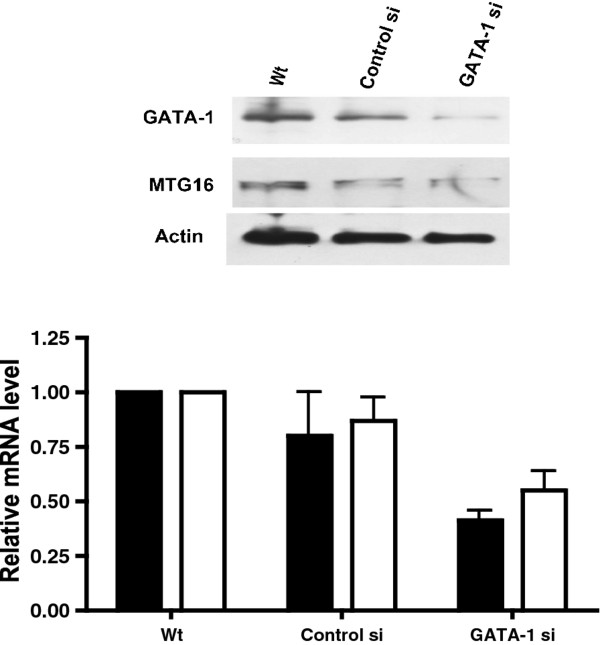
**Effects on the *MTG16 *promoter activity of GATA-1 siRNA**. Transfection was as described in Methods. The relative mRNA expression of GATA-1 (black bar) and MTG16 (white bar) is given for HEL cells (wt), HEL cells transfected with non silencing scrambled control siRNA (control si) and HEL cells transfected with siRNA for GATA-1 (GATA-1 si**)**. The protein expression of GATA-1 and MTG16 are shown with GAPDH as acontrol of equal loading. The experiments were performed in triplicate. Bars represent the mean and the error bars show SEM.

**Figure 10:**
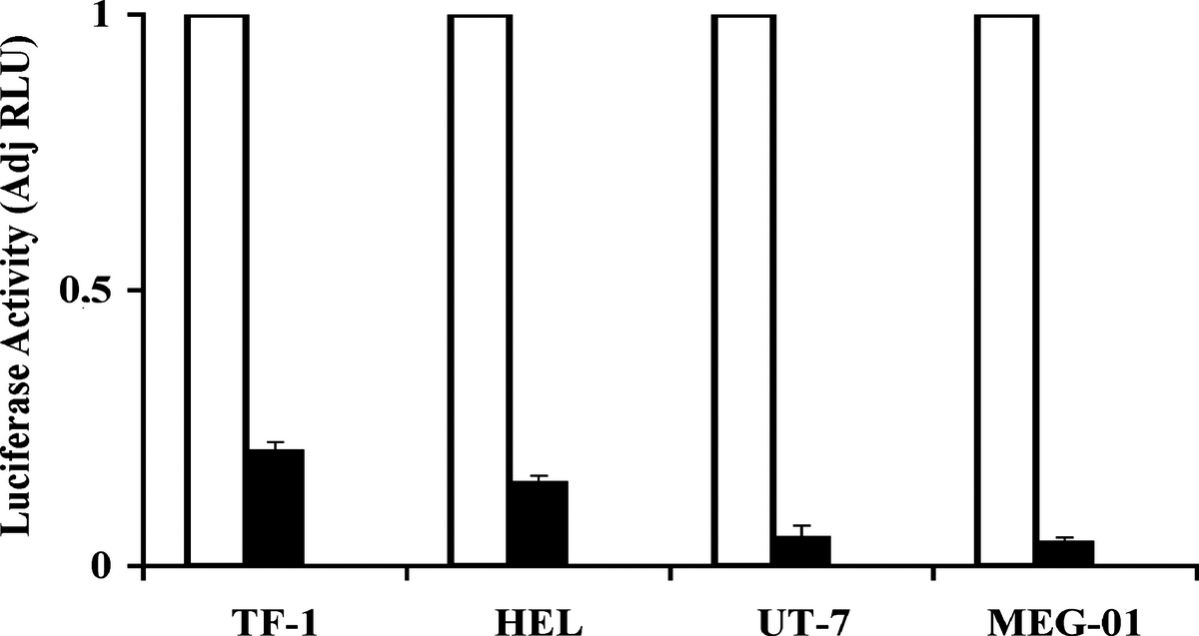
**Inhibition of the *MTG16 *promoter activity through inhibition of GATA-1 by HERP2**. TF-1, HEL, UT-7, or MEG-01 cells were co-transfected with 10 μg MTG16 -668 to -57 bp promoter and the HERP2 plasmid construct. White bars show MTG16 -668 to -57 bp promoter activity without HERP2. Black bars show activity after co-transfection with HERP2, relative to the luciferase activity of the MTG16 -668 to -57 bp promoter in the absence of HERP2 (white bars). All cell lines examined demonstrated repression of GATA-1 driven MTG16 luciferase promoter. Renilla was not affetected by HERP2. The experiments were performed in triplicate. Bars represent the mean and the error bars show SEM.

To verify a role of GATA-1 in *MTG16 *expression, siRNA was transfected into HEL cells as described in Methods. Introduction of GATA-1 siRNA led to a decrease by approximately 50% of both GATA-1 and MTG16 mRNA levels and the results were confirmed on the protein levels (Figure 9), indicating a role of GATA-1 in *MTG16 *transcriptional regulation. Three different siRNA constructs, all bioinformatically (Rosetta algorithm, SIGMA) designed to minimize off-target effects were used. However, only one resulted in a robust suppression of GATA-1 protein. Off-target effects can therefore not be excluded. However, in combination with our results from reporter experiments, EMSA, ChIP, and overexpression, we believe that we can draw the conclusion that GATA-1 is a positive trans-acting factor on the promoter. Additional support for a role of GATA-1 was obtained by use of the basic helix-loop-helix transcription factor HERP2, which shows physical interaction with GATA-1 and represses GATA-1-mediated transcriptional activation [29]. Both erythroid and megakaryocytic cell lines cotransfected with HERP2 showed strong inhibition of the *MTG16 *promoter reporter (Figure 10) consistent with a role of GATA-1 in transcriptional activation of *MTG16*.

### Functional evaluation of the *MTGR1 *promoter

**Figure 11:**
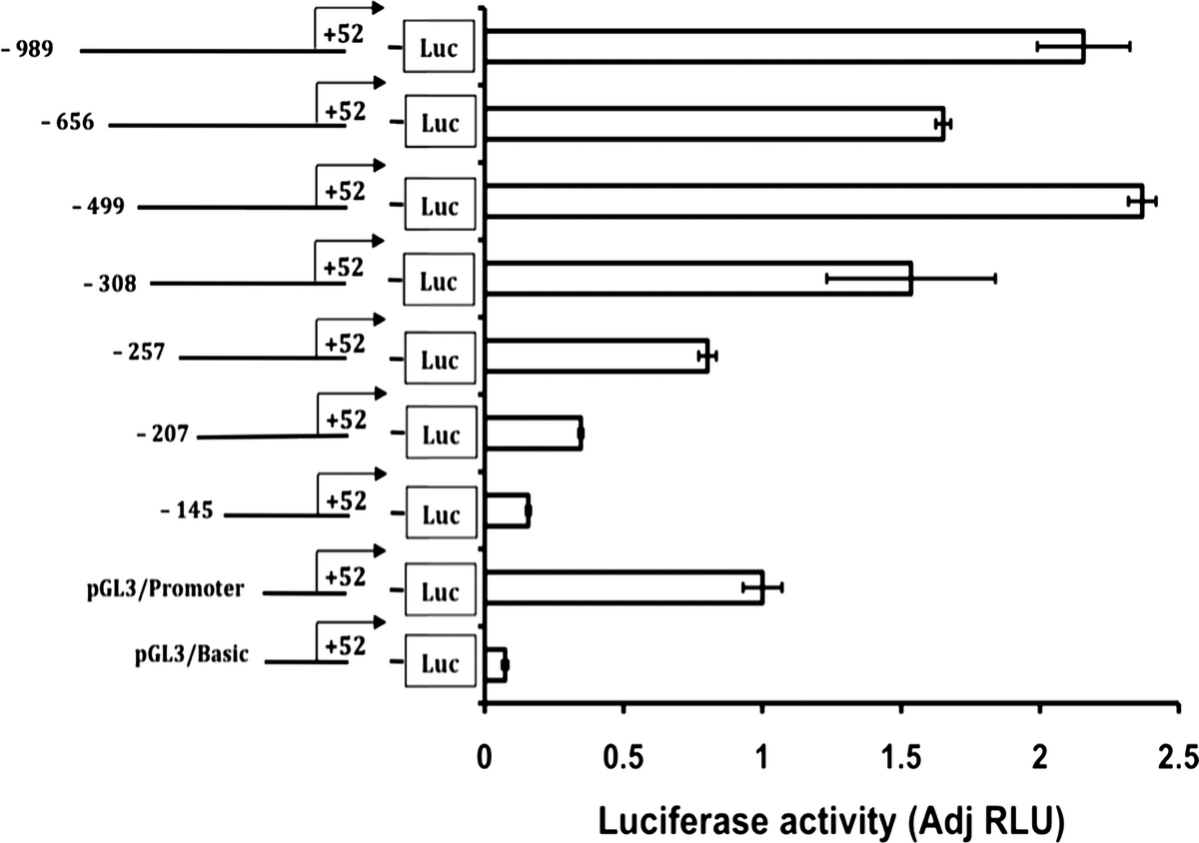
**Reporter gene analysis of the *MTGR1 *promoter**. *Functional analysis by sequential 5'-deletion*. The 5'deleted *MTGR1 *reporter constructs -989 to +52 (-989), -656 to +52 (-656), -499 to +52 (-499), -308 to +52 (-302), -257 to +52 (-257), -207 to +52 (-257), and -145 to +52 (-145) were examined after expression in HEL cells. The transcriptional activity is retained by the -499 to +52 bp region indicating the location of the proximal *MTGR1 *promoter. The pGL3/basic and pGL3/SV40-promoter are used as negative and positive control, respectively in (A) and (B). The luciferase activity was normalized against pGL3/SV40-promoter activity and is shown for 3 to 5 separate transfections; bars represent the mean and the error bars show SEM.

**Figure 12:**
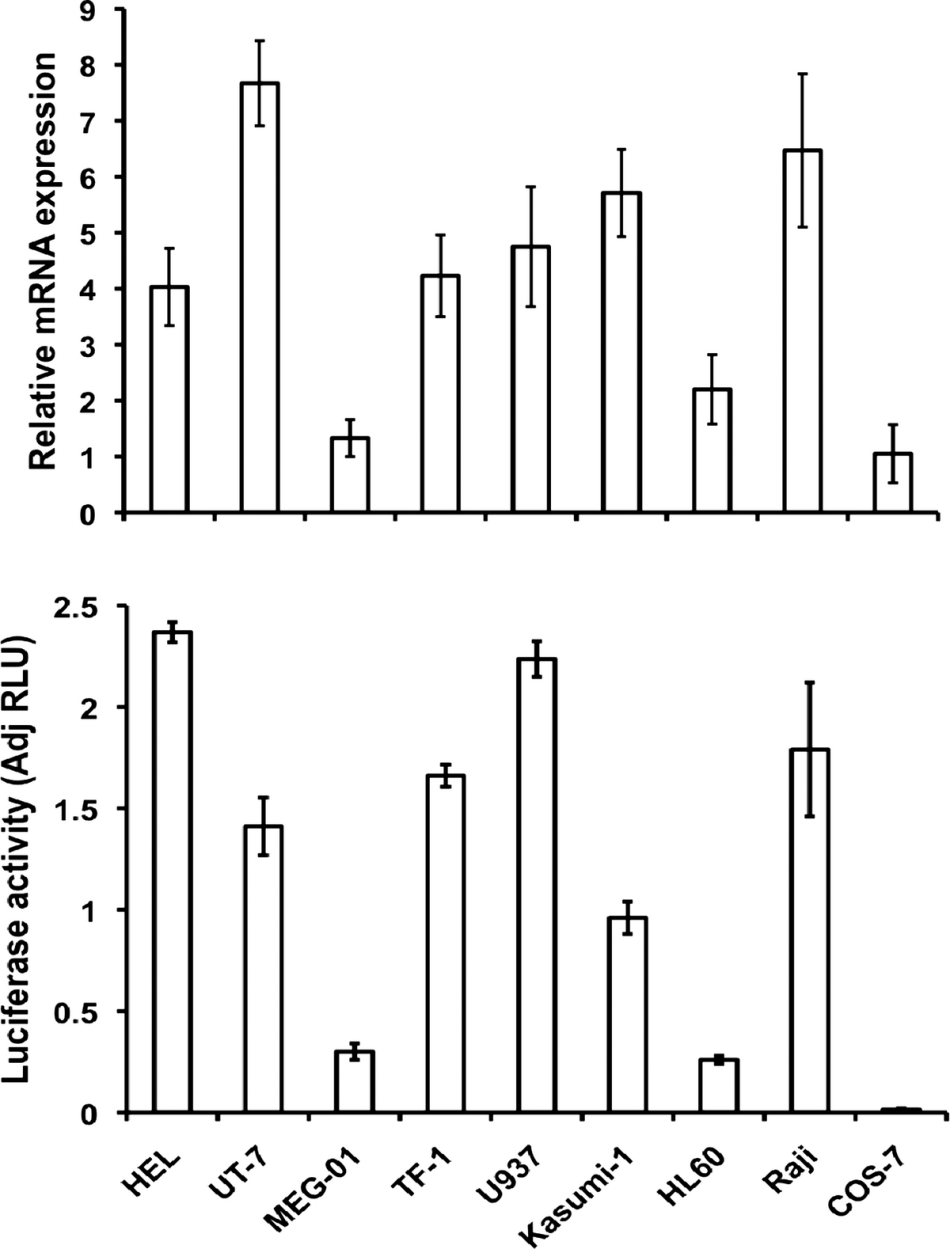
***MTGR1 *promoter activity in various cell lines**. pGL3/-499 to +52 (the shortest construct with full promoter activity) was transfected to indicated cell lines and luciferase activity was determined as described in Methods. The relative *MTGR1 *mRNA expression of the cell lines is also given. The promoter shows a 1 to 2.5-fold increased reporter signal in most cell lines. MEG-01, HL-60 and COS cells show both the lowest reporter signals and the lowest *MTGR1 *mRNAs.

We cloned a -989 to +52 bp region (translational start codon at +1) for examination of transcriptional activity. The cloned region was inserted upstream of the luciferase reporter gene in promoterless pGL3/Basic vector to give the pGL3/-989 to +57 plasmid, which was transfected into hematopoietic cell lines and luciferase activity was determined and normalized as described in Methods. An approximately 2-fold luciferase signal in HEL cells, as compared to the positive control pGL3/promoter, indicated strong transcriptional activity of the cloned region (data not shown). Upon transfection of the shortest luciferase construct with full promoter activity (pGL3 -499 to +52 bp) (see Figure 11 below) to a panel of hematopoietic cell lines, different activities were revealed (Figure 12). As with the MTG16 promoter, the strongest activity was found in HEL cells, but the activity patterns between the two promoters were not uniform (compare Figure 3 to Figure 12). The *MTGR1 *promoter construct produced a signal in all cell lines examined except for COS cells. All cell lines examined expressed endogeneous *MTGR1 *mRNA supporting ubiquitous expression (Figure 12).

To define the promoter in more detail, a series of constructs was generated by sequential deletions from the 5'end of the -989 +52 bp region after which transcriptional activity was analyzed in HEL cells (Figure 11). Deletion to -499 did not affect transcriptional activity, but further deletion reduced the luciferase reporter expression. Thus the sequence from -499 to +52 bp retained full transcriptional activity and therefore is likely to contain the core proximal *MTGR1 *promoter. Further sequential 5' deletions of the -499 to +52 bp region resulted in a step by step decreasing luciferase reporter signal (Figure 11) suggesting cooperation between factors binding to different regions of the promoter.

Site-directed mutagenesis was used to examine whether potential transcription factor binding sites contributed to promoter transactivation. The -308 to +52 bp region retains approximately two thirds of the transcriptional activity of the fully active promoter as was shown in Figure 11. This region is GC box-rich with 9 SP1 consensus binding sites (Figure 2) located immediately upstream of the transcription start site at -146 bp consistent with the housekeeping gene character of *MTGR1 w*ith ubiquitous expression. However, disruption of individual SP1 sites; SP1-285, SP1-243, SP1-229, SP1-225, SP1-213, SP1-206, SP1-192, SP1-178, SP1-170; did not affect reporter gene activity relative to intact *MTGR1 *promoter (data not shown), suggesting that SP1 is not important for promoter activity. However, it is possible that multiple active SP1 binding creates a redundancy in SP1 binding and elimination of several SP1 binding sites is needed to reveal their functional relevance. Disruption of the -335 ETS1, the -276 ETS1 and the -301 YY1 sites did also not affect promoter activity (data not shown). Overall, our results indicate that a GC-box-rich sequence with multiple SP1 sites is important for transcriptional regulation of MTGR1 promoter.

### AML1-ETO represses *MTG16 *and *MTGR1 *gene reporters

**Figure 13:**
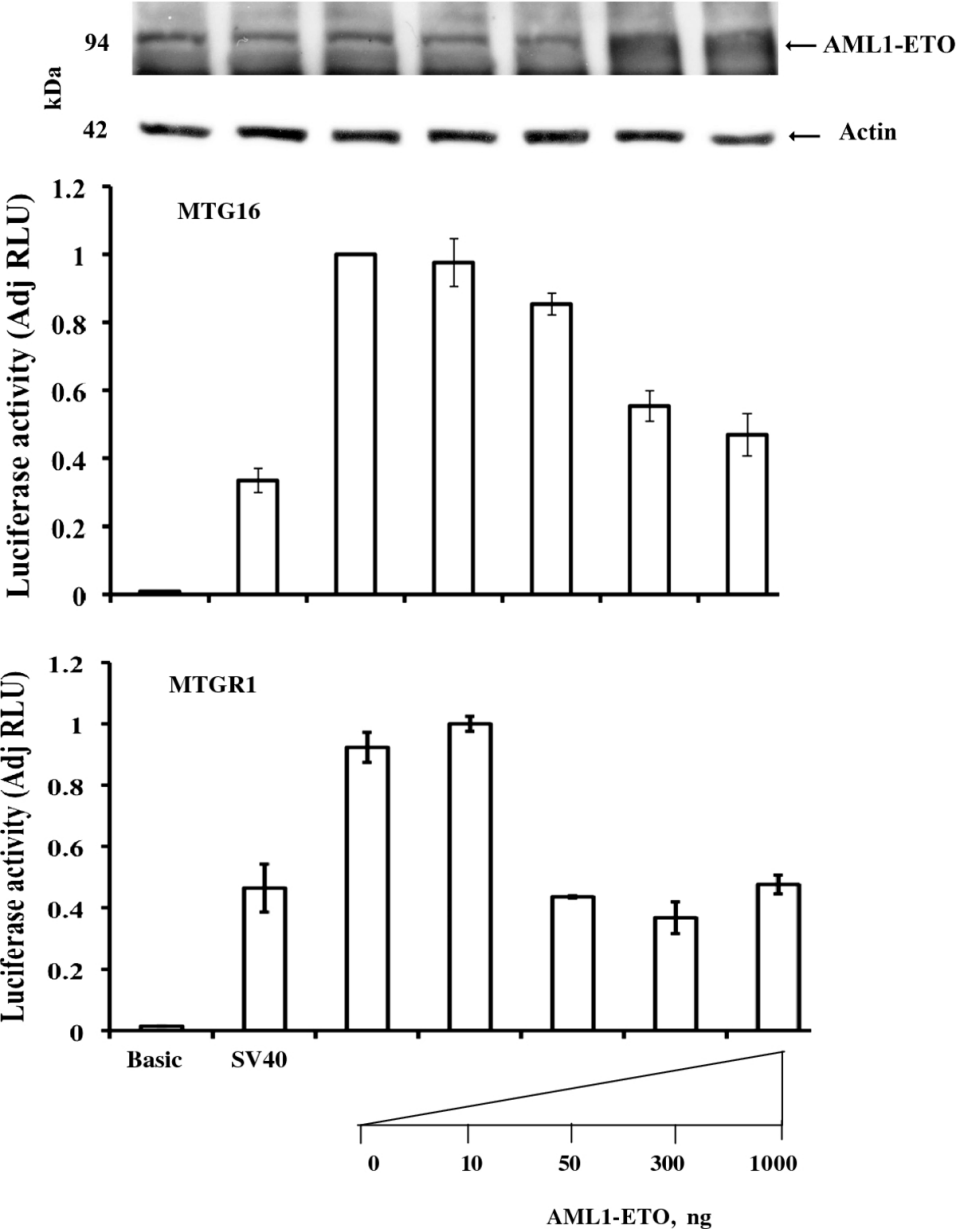
**Effects of AML1-ETO on the *MTG16 *and *MTGR1 *promoter reporter**. Erythroid HEL cells were co-transfected with 15 μg *MTG16 *-668 to -57 bp promoter plasmid (-668) or *MTGR1 *-499 to +52 bp promoter plasmid (-499) and 0 to 1 μg of *AML1- ETO *plasmid. pGL3/Basic (Basic). pGL3/SV40 (SV40). The luciferase activity is normalized against *MTG16 *-668 to -57 bp or *MTGR1 *-499 to +52 bp promoter. Both promoters are repressed in a dose-dependent manner by AML-ETO. Western blotting shows exogeneous AML1-ETO expression detected with anti-MTG. The experiments were repeated three times with similar results.

The AML1-ETO fusion protein, which is a gene product of t(8;21) of acute leukemia [14,15] binds the promoter region of many genes causing transcriptional suppression [30]. AML1- ETO has been shown to suppress an *ETO *promoter reporter in erythroid/megakaryocytic cells [25]. Therefore, AML1-ETO was transiently expressed in HEL cells to examine also the effect on co-transfected *MTG16 *and *MTGR1 *promoter reporters. In both cases the reporter was strongly repressed in a dose-dependent manner by AML1-ETO (Figure 13). This finding is not directly consistent with the inhibition observed *in vivo *in AML1-ETO-positive Kasumi cells of only *MTG16 *(Figure 3) but not *MTGR1 *transcripts (Figure 12) suggesting influence of cell-specific contexts.

## Discussion

The negative transcriptional regulator ETO was originally discovered as the 3' participant in the leukemia chromosomal translocation t(8;21) [31]. Now, ETO and its homologues MTG16 and MTGR1 are known to be involved in transcriptional regulation of genome-wide targets. To explain cell-type-specificity of ETO homologue expression and function [18] exploration of promoter regulation is warranted. The human *ETO *proximal promoter is regulated by the GATA-1 transcription factor in an erythroid/megakaryocytic-specific manner [25]. Here, we continued to identify essential *cis*-acting elements and *trans*-acting factors also of the promoters of *MTG16 *and *MTGR1 *within human hematopoietic cells.

### *MTG16 *promoter regulation

TATA and CAAT-like promoter motifs facilitate interactions with general transcription factors and are thus involved in initiation of transcription. The proximal *MTG16 *promoter investigated lacks these motifs. GC-boxes are known to regulate the utilization of start sites in TATA-less promoters [32-35]. Therefore, the conserved GC-box situated close to start site (Figure 1A) may be involved in transcriptional initiation. Among hematopoietic cell lines, the promoter activity was strongest in the erythroid HEL/TF-1, erythroid/megakaryocytic UT-7 and megakaryocytic MEG-01 cells consistent with a role of MTG16 in erythropoiesis/megakaryopoiesis [5,8,20,21]. Deletion analysis of the proximal *MTG16 *promoter defined a 492 bp region upstream of the transcription start to be required for maximal promoter activity. This region includes a potential GATA binding site (-301), which is strongly conserved between species. Disruption of the GATA -301 binding site, repressed transactivation of the *MTG16 *promoter. The importance of this *cis*-acting binding site was confirmed by results from examination *in vitro *with EMSA, which revealed specific GATA-1 binding. Further, in vivo binding of both GATA-1 and GATA-2 to this region of the promoter was confirmed by ChIP/PCR analysis. Taken together these results demonstrate binding of the GATA-1 transcription factor to the *MTG16 *promoter and strongly support a role for GATA-1 in transcriptional regulation of the *MTG16 *gene in hematopoietic cells.

### *MTGR1 *promoter regulation

A 499 bp region upstream of the translation start site of *MTGR1 *was defined as sufficient for maximal activity in erythroid HEL cells. Upon analysis using different cell lines, we found that the promoter is active in most of them, which is in agreement with ubiquitous presence of native *MTGR1 *gene product. Furthermore, the lack of classical TATA or CCAAT boxes, the presence of multiple SP1 sites and a CpG island covering the transcriptional initiation site of the *MTGR1 *promoter (Figure 2) are similar to characteristics of a housekeeping gene [36], expected to be generally active in a non-tissue specific manner. Few potential binding sites for known transcription factors were found in the -499 promoter, rather a GC-box-rich sequence stretch with multiple SP1 binding sites is a prominent part of the promoter. Importantly, the GC-box-rich region was responsible for most of the transcriptional activity. However, we were not able to inhibit promoter activity by the elimination of individual SP1 binding sites. Possibly, individual SP1 binding sites may be equally involved in guaranteeing constitutive *MTGR1 *expression. Further 5'deletion of the -499 promoter region showed gradually decreasing promoter activity observed upon successive deletions, indicating transcriptional cooperation between promoter sequences consistent with cooperating SP1- binding sites.

### ETO homologues in hematopoiesis

The regulation of both the *ETO *[25] and the *MTG16 *promoter (this work) by GATA-1 is consistent with a role of human ETO/MTG16-mediated gene suppression at a phase of erythropoiesis/megakaryopoiesis when GATA-1 levels are high [37,38]. However, despite shared *trans*-activation, *MTG16 *and *ETO *are differently expressed in human hematopoiesis [18]; *MTG16 *is downmodulated at late stages of erythropoiesis whereas *ETO *is expressed transiently in (human) erythropoiesis. Therefore, cell-type-specific expression of these ETO homologues may be governed also by *trans*-activating factors other than GATA. Although both *MTG16 *and *ETO *are expressed in human erythroid cells [18] yet the role of ETO in murine erythropoiesis is not yet supported [10].

GATA-1 and GATA-2 are expressed reciprocally during development of the erythropoietic lineage, GATA-2 decrease is followed by GATA-1 increase [39]. GATA-2 seems to be essential for the development of early and definitive erythropoiesis/megakaryopoiesis and GATA-1 for terminal erythroid/megakaryocytic maturation [26-28]. The present results are consistent with upregulation of MTG16 as a result of rising GATA-1 levels. Furthermore, results from genome-wide analyses [40] are consistent with early GATA-2 induction of murine ETO-2 during erythropoiesis. These analyses also showed that most occupied sites are located away from promoters indicating that GATA factors may be involved in long- range control by occupancy at non-promoter regions.

Our finding of *in vitro *binding of GATA-1 but lack of GATA-2 binding to the GATA -301 binding site raises questions about the relative importance of GATA-1/GATA-2 in *MTG16 *promoter regulation. GATA-2 is reported to contribute in hematopoietic stem cell and progenitor survival [41] and GATA-1 in erythroid cell survival [42,43]. Furthermore, genetic complementation studies show that GATA-1 can replace GATA-2 [44,45]. A lack of binding in EMSA experiments suggests that GATA-2 may not be a primary transcription factor to initiate nucleation of the transcription complex at the *MTG16 *promoter. Possibly, GATA-2 has a weaker binding affinity than GATA-1. However, our finding in ChIP assay of the binding of both GATA-1 and GATA-2 to *MTG16 *promoter suggests that GATA-1 and GATA-2 cooperate. This is supported by results from *in vivo *studies by ChIP-seq with human K562 erythroleukemia cells [40] showing chromatin occupancy by both GATA-1 and GATA-2 at the conserved GATA motif corresponding to the -301 site of the *MTG16 *promoter. In addition, a role of GATA-2 in stimulating murine *ETO-2 *expression was also suggested [40]. Thus, GATA-2 was found to occupy a conserved GATA binding site in the murine erythroid G1E cell, which lack GATA-1 expression; the occupied murine site corresponds to the human -301 GATA binding site of *MTG16 *promoter. Overall, available data suggest a role of both GATA-1 and GATA-2 in *MTG16 *promoter activation.

Murine MTG16 (ETO2) is involved in erythroid progenitor expansion [5], which is coupled to inhibition of differentiation. This is also the case in megakaryopoiesis where ETO2 represses inappropriate early expression of terminal genes [22] coupled to restrained differentiation of immature megakaryocytes. *MTG16 *and *ETO *show reciprocal expression during *in vitro*-induced human erythropoiesis; MTG16 decreases and ETO increases during the peak of erythropoiesis [18]. As both promoters are regulated by GATA-1, specific negative autoregulation of *MTG16 *[40] may explain the difference. This is consonant with a role for MTG16 in repressing genes associated with terminal erythroid differentiation thereby inhibiting differentiation and a role for ETO in repressing genes associated with self-renewal and proliferation thereby supporting erythroid differentiation.

### Suppression of *MTG16/MTGR1 *promoter by AML1-ETO

The leukemic AML1-ETO fusion protein [14,15] binds to the promoter region of a number of genes resulting in transcriptional suppression [30]. However, some genes are affected by AML1-ETO in an indirect manner and do not bind the fusion protein directly [30,46]. The observed suppression of the *MTG16 *and the *MTGR1 *promoter by AML1-ETO is likely to be indirect as binding sites for AML1 were not detectable on the promoters. Furthermore, a potential mechanism for the *MTG16 *promoter inhibition by AML1-ETO would be suppression of *GATA-1 *through impaired acetylation [47]. Suppression of *MTG16 *is consistent with the low *MTG16 *promoter reporter signal observed in *AML1-ETO*-positive Kasumi cells, which showed very low *MTG16 *transcripts (Figure 3), whereas *MTGR1 *transcripts were high in this cell line (Figure 12). AML1-ETO-mediated suppression of the *ETO *promoter has also been reported [25] and suggested to facilitate the AML1-ETO- induced block of erythroid lineage commitment [47,48]. AML1-ETO-mediated repression of *MTG16 *may have a similar effect because of blocking MTG16/ETO2-mediated erythroid progenitor expansion [5]. The repression of all *ETO *homologue genes by AML1-ETO may have a general role in inhibition of hematopoietic differentiation in t(8;21) leukemia.

## Conclusions

This work shows differences in promoter regulation between *MTG16 *and *MTGR1 *that may explain differences in hematopoietic expression of these genes. Evolutionary conserved GATA binding sites are critical in transcriptional regulation of both *MTG16 *(this work) and *ETO *[25] promoters. The *MTGR1 *promoter has a GC box-rich sequence with multiple SP1 binding sites, which may secure transcriptional regulation and ubiquitous expression of this gene.

## Methods

### Cell Culture

Myelomonocytic U-937, erythroid HEL and TF-1, megakaryocytic MEG-01 cells were grown in RPMI-1640 medium supplemented with 10% Fetal Bovine Serum (FBS) (Gibco BRL, Life Technologies, Rockville, MD, USA); TF-1 cells also received 20 ng/ml GM-CSF (R&D Systems, MN, USA). The erythroid/megakaryocytic UT-7 cells were grown in MEM Alpha modification medium with L-Glutamine and Nucleosides (PAA Laboratories GmBh, Austria) supplemented with 20% FBS and 30 ng/ml GM-CSF. The acute myeloid leukemia Kasumi-1, Burkitt's lymphoma Raji, and promyelocytic HL-60 cells were supplemented with 20% FBS. Monkey kidney COS cells were grown in DMEM medium with 10% FBS supplemented with high glucose (4.5 g/L) and L-glutamine.

### Identification of the transcription start site of the *MTG16 *gene

The 5'-end (transcription start) of the mRNA was identified by 5'-RACE carried out with mRNA prepared from HEL cells using Oligotex Direct mRNA mini Kit (Qiagen, Hilden, Germany), using the First choice RNA Ligase mediated (RLM)-RACE kit (Ambion Inc., TX, USA). Nested PCR of RACE reactions was performed with adapter primer 5'-CGCGGATCCGAACACTGCGTTTGCTGGCTTTGATG-3' and nested gene specific primer 5'-GCCTTAGCTTTCCTGTCCACTGG-3'. RACE- products were cloned into pGEM-T Easy Vector system (Promega Corporation, WI, USA) and sequenced.

### Amplification of *MTG16 *promoter region

A 1208 bp region upstream of the translation start (from -1264 to -57 bp) was amplified from human genomic DNA by PCR. The forward and reverse primers used were 5'-TGAGGCGGTACCACCTCCAGCCATGGCATC -3' and 5'-TGCCTAGATCTACCTCCTGCAGCCTTGAGG -3', respectively. Regions corresponding to -905 to -57 and -820 to -57 bp were amplified from the 1208 bp fragment by nested PCR with forward primers 5'- TAT***GGTACC***GTGGGGGAGCCCTGCTGTCTCCACA -3' (KpnI restriction site underlined),5'- AGGCGGGA**GGTACC**TTGAGGACAGGTCAGG -3' (KpnI restriction site underlined), and a common reverse primer 5'-TGCCT**AGATCT**ACCTCCTGCAGCCTTGAGG -3' (BglII restriction site underlined).

Sequential 5' deletions of the -820 to -57 bp promoter region were generated by PCR from the cloned genomic DNA as template to generate -668 to -57, the -512 to -57, the -359 to -57 bp, the -339 to -57, the -299 to -57 and the -219 to -57 regions. Forward primers were 5'-*TA*A***GGTACC***CTGGGGAAAGCTCGGCTGAC-3', 5'-TAT***GGTACC***CCTGCCCTGGGGCCGAGG -3', 5'-CCA***GGTACC***TCCTGTCAGGGAAGTGGCGG-3', 5'-TATGGTACCGGGCGCAGCTGCAGGCC

-3', 5'- TAT***GGTACC***TCACGGGGACACAGCTGGC-3' and 5'-TAT***GGTACC***AT*G*AGCCCCAGGGCTCCCACCC -3' with KpnI restricton sites underlined. The common reverse primer is the same as used for amplification of the -1208 to -57 bp region. All sequences were verified by sequencing.

### Amplification of *MTGR1 *promoter region

A 5' flanking region of 1965 bp from -1913 to +52 bp was amplified from human genomic DNA by PCR. The forward and reverse primers used were 5'-TATTGGTACCGCAACACCATGTCTGGCTAA -3' (KpnI restriction site underlined) and 5'- CTACAGATCTGCATTCACGCCCCACTTAC -3' (BglII restriction site underlined), respectively. Regions corresponding to -989 to +52, -656 to +52 and -499 to +52 bp were amplified from the 1965 bp fragment by nested PCR with forward primers 5'-TTGCGGTACCCTTCAAACTCCTGACCTCGTGA -3' (KpnI restriction site underlined), 5'- TTGCGGTACCCCCGGCCAACAGTTATTAAA -3' (KpnI restriction site underlined), 5'- TTGCGGTACCTGTAATCCCTCCCCCTAGCACT -3' (KpnI restriction site underlined), and a common reverse primer 5'-CTACAGATCTGCATTCACGCCCCACTTAC -3' (BglII restriction site underlined).

Sequential 5' deletions of the -499 to +52 bp promoter region were generated by PCR from the cloned genomic DNA as template to generate -308 to +52, the -257 to +52, the -207 to +52 bp and the -145 to +52 regions. Forward primers were 5'-TTGCGGTACCGTGCTTAATGGGAGCTGGTCT.-3', 5'-TTGCGGTACCGCGGCGAGAGCGCGCGGCGGGGGCG -3', 5'-TTGCGGTACCGCCGGGGGCGGGGCGGGGCGCGGCC -3' and 5'-TTGCGGTACCGTGGTGGTGTCTGGTTAGCTC-3', with KpnI restricton sites underlined. The common reverse primer is the same as used for amplification of the -499 to +52 bp region. All sequences were verified by sequencing.

### Site-directed mutagenesis of transcription factor-binding sites

Oligonucleotide primers including the desired mutations were synthesized and used in two-step spliced overhang extension PCR. The following potential *MTG16 *promoter transcription factor binding sites were mutated: ETS1/PU.1 sites at positions -491, 5'-A**GGA**AA-3' changed to 5'- A**TTC**AA-3'; SP1 sites at positions -425, 5'-G**GGA**GG-3' changed to 5'-G**TTC**GG-3'; ETS1/SPI1 sites at positions -419, 5'-A**GGA**AGT-3' changed to 5' - A**TTC**AGT-3' TFAP2A sites at positions -389, 5'-GCC**CGA**GGG-3' changed to 5'-GCC**TTC**GGG-3'; GATA/YY1 sites at positions -356, 5'-GA**TGG**T-3' changed to 5'-GA**CTT**T-3'; SP1 sites at positions -316, 5'-CTCC**GGC**CCC-3' changed to 5'-CTCC**TTA**CCC-3'; GATA sites at positions -301, 5'-T**ATC**A-3' changed to 5'-T**GGT**A-3' and MZF1 sites at positions -251 & -191, 5'-T**CCC**CA-3' changed to 5'-T**AAA**CA-3'.

The following potential *MTGR1 *promoter transcription factor binding sites were mutated: ETS1 sites at positions -335, 5'-C**TTCC**G-3' changed to 5'-C**GTAT**G-3'; YY1 sites at positions -301, 5'-C**CCA**TT-3'changed to 5'-C**TTC**TT-3'; SP1 sites at positions -285, 5'-C**C**C**G**G**C**CCCA-3' changed to 5'- C**A**C**T**G**T**CCCA -3'; ETS1 sites at positions -276, 5'-C**T**T**CC**G-3' changed to 5'- C**G **T**AT**G -3' SP1 sites at positions -243, 5'-CCC**CC**GC**C**GC-3' changed to 5'- CCC**TT**GC**A**GC -3'; SP1 sites at positions -229, 5'-CC**CC**GC**C**CGC-3' changed to 5'- CC**TT**GC**A**CGC -3'; SP1 sites at positions -225, 5'-CC**CC**GC**C**CCG-3' changed to 5'- CC**TT**GC**A**CCG -3'; SP1 sites at positions -213, 5'-CC**CC**GC**C**CCC-3' changed to 5'- CC**TT**GC**A**CCC -3'; SP1 sites at positions -206, 5'-CC**CC**GC**C**CCG-3' changed to 5'- CC**TT**GC**A**CCG -3'; SP1 sites at positions -192, 5'-CG**CC**GC**C**GCC-3' changed to 5'- CG**TT**GC**A**GCC -3'; SP1 sites at positions -178, 5'-CG**CC**GC**C**GCC-3' changed to 5'- CG**TT**GC**A**GCC -3' and SP1 sites at positions -170, 5'-CG**CC**GC**C**GCC-3' changed to 5'- CG**TT**GC**A**GCC -3'. After subcloning into promoterless pGL3/Basic reporter plasmid, all mutations were verified by sequencing.

### Luciferase reporter assays of *MTG16 *and *MTGR1 *promoter

*MTG16 *promoter was cloned into promoterless pGL3/Basic reporter plasmid using firefly luciferase as reporter to generate pGL3 -905-57, pGL3 -820-57, pGL3 -668-57, pGL3 - 512-57, pGL3 -359-57, pGL3 -339-57, pGL3 -299-57 and pGL3 -219-57 reporter constructs. Mutants were cloned into the same reporter plasmid. Transient transfections of hematopoietic cell lines were performed by electroporation as previously described by [49]. The pGL3/SV40-promoter vector served as positive control and promoterless pGL3/Basic vector as negative control. Renilla luciferase, pRL/SV40 vector (Promega Corporation, WI, USA) was used as internal control for transfection efficiency. Thirtyfive μg pGL3 DNA were used for HL-60 target cells and 15 μg for HEL, TF-1, U937, UT-7, MEG-01, Raji and Kasumi-1 target cells. COS cells were transfected with 1.5 μg of DNA by use of Polyfect (Qiagen, Hilden, Germany). Twentyfour hours after transfection, cells were lysed and subjected to luciferase dermination using the Dual luciferase reporter assay kit (Promega Corporation, WI, USA). Firefly and Renilla luminiscense were quantified (Run Promega Protocol, DLR-0-INJ) in a GLOMAX 20/20 Luminometer (Promega Corporation, WI, USA). Firefly was normalized to Renilla luciferase as internal control for transfection efficiency. Results are given as adjusted Relative Luciferase Units (AdjRLU) normalized to pGL3/promoter (set to 1). Three to five independent transfections were performed in each case and each sample was measured in triplicate.

Similarly, *MTGR1 *pGL3 -1913 + 52, pGL3 -989 + 52, pGL3 -656 + 52, pGL3 -499 + 52, pGL3 -308 + 52, pGL3 -257 + 52, pGL3 -207 + 52 and pGL3 -145 + 52 reporters were generated, transfected into cells and assayed as described above.

### Transfection of GATA-1 small interfering RNA

Twentyfour-well Falcon plates (Becton Dickinson, New Jersey 07417, USA) were coated with 1 mg/ml retronectin (Takara Bio inc, Verviers, Belgium) in PBS for two h at room temperature. Excess retronectine was removed and wells were blocked with 2% BSA in PBS for 30 min. After removal of the supernatant, 50,000 HEL cells were added per well in 600 μl RPMI medium containing 10% FBS and 20 nM phorbol 12-myristate 13-acetate (PMA) to generate adherent cells by incubation at 37°C for 24 h. After washing with PBS transfection was carried out with 5 μM siRNA using the N-TER transfection reagent (Sigma-Aldrich Corp. St. Louis, MO USA) in RPMI without FBS. After 4 h, 300 μl of RPMI with 20% FBS was gently added and cells were incubated for 48 h. Eventually, gently rinsed with PBS, tripsinized and examined by Western blotting. The GATA-1 siRNA sequence was 5'-AGUUGAGGCAGGGUAGAGC-3' (Oligo SASI_Hs02_00333089, Sigma).

### Inhibition of GATA-1 transcriptional activity by HERP2

The transcription factor HERP2 represses transcriptional activation by GATA-1 [29] by physical interaction. To get support for a role of GATA-1 in gene activation of *MTG16*, HEL, TF-1, UT-7, and MEG-01cells were co-transfected with 10 μg MTG16 pGL3 -668 luciferase reporter and 10 μg HERP2 plasmid (kindly provided by Dr N Goldfarb, Charlottesville, Virginia) and the reporter signal was measured after 24 h as described above for luciferase reporter assays. The results were normalized to the luciferase activity of pGL3 -668 luciferase reporter.

### Electrophoretic Mobility Shift Assay (EMSA) of *MTG16 *promoter

EMSA was performed as described previously [25]. The potential GATA-1 sites at -301 was examined. The probe sequence was biotin-5-CCCGGCATTATCACGGGGACAC -3'. Nuclear extracts from HEL, TF-1, UT-7 and MEG-01 cells were prepared as described by Andrews and Faller [50]. One to two μl polyclonal anti-GATA-1 (Active Motif, Carlsbad, CA, USA), monoclonal anti-GATA-2 (Santa Cruz Biotechnology Inc., CA, USA, sc-9008) or polyclonal anti-CD63 (sc-7080, Santa Cruz Biotechnology Inc., CA, USA) antibodies were added to the reaction mixtures.

### Chromatin Immunoprecipitation (ChIP) assay of *MTG16 *promoter

ChIP was performed as described previously [25]. For IP, 4 μl of polyclonal anti-GATA-1 (Active Motif, Carlsbad, CA, USA) or 0.8 μg monoclonal anti-GATA-2 antibodies (R&D Systems, MN, USA) were used. Forward and reverse primers for GATA sites were 5'-CAGATGGTTCCTGTCAGGGAAGTGGCG-3' and 5'-AGGTCTCCCTGCAGCCTGCGGGTGAG-3'. Control forward and reverse primers were: 5'-TAACACAGAGTACCCAGCCACTGTGC-3 ' and 5'-TGGTTGCACGGACAGAAGCCCCT-3'. Three different chromatin preparations were used for each IP.

### Quantitative real-time PCR

Real-time PCR was performed as described previously [18]. Transcript levels were calculated from a standard curve based on the Ct values of the samples,. Relative quantification based on the ΔCt method [51] was used. Normalization: ΔCt = Ct (sample) - Ct (HEL corresponding DNA concentration). Relative quantification = 2 **^- ΔCt ^**.

### Immunoprecipitation (IP) and Western blotting

IP and Western blotting were performed as described previously [52]. The following antibodies were used: polyclonal anti-GATA-1 (Active Motif, Carlsbad, CA, USA) and polyclonal anti-GATA-2 (R&D Systems, MN, USA).

### Bioinformatics

The cDNA sequences were analyzed by use of the NCBI Blast program http://www.ncbi.nlm.nih.gov/BLAST/. Conserved regions were searched by multiple alignment to genomic sequences using ClustalW http://www.ebi.ac.uk/Tools/clustalw2/index.html.

Potential transcription factor binding sites were examined with the MatInspector http://www.genomatix.de//matinspector.html and the Jaspar database (Jaspar.genereg.net).

### Statistical analysis

The statistical significance between two samples was determined by unpaired t-test in Prism Programme. Triple and single asterisk represents *P *< 0.0001 and *P *< 0.05, respectively.

## Competing interests

The authors declare that they have no competing interests.

## Authors' contributions

RA carried out experiments, analyzed data, supervised experimental design/data analysis and drafted the manuscript. PK carried out experiments, analyzed data and drafted the manuscript. RSD supervised experimental design/data analysis. UG supervised experimental design/data analysis. IO supervised experimental design/data analysis and drafted the manuscript. All authors critically revised and approved the final manuscript.

## References

[B1] Feinstein PG Kornfeld K Hogness DS Mann RS Identification of homeotic target genes in Drosophila melanogaster including nervy, a proto-oncogene homologue Genetics 1995 140 2 573 586 7498738 10.1093/genetics/140.2.573 PMC1206636

[B2] Gelmetti V Zhang J Fanelli M Minucci S Pelicci PG Lazar MA Aberrant recruitment of the nuclear receptor corepressor-histone deacetylase complex by the acute myeloid leukemia fusion partner ETO Mol Cell Biol 1998 18 12 7185 7191 9819405 10.1128/mcb.18.12.7185 PMC109300

[B3] Lutterbach B Westendorf JJ Linggi B Patten A Moniwa M Davie JR Huynh KD Bardwell VJ Lavinsky RM Rosenfeld MG ETO, a target of t(8;21) in acute leukemia, interacts with the N-CoR and mSin3 corepressors Mol Cell Biol 1998 18 12 7176 7184 9819404 10.1128/mcb.18.12.7176 PMC109299

[B4] Chevallier N Corcoran CM Lennon C Hyjek E Chadburn A Bardwell VJ Licht JD Melnick A ETO protein of t(8;21) AML is a corepressor for Bcl-6 B-cell lymphoma oncoprotein Blood 2004 103 4 1454 1463 14551142 10.1182/blood-2003-06-2081

[B5] Goardon N Lambert JA Rodriguez P Nissaire P Herblot S Thibault P Dumenil D Strouboulis J Romeo PH Hoang T ETO2 coordinates cellular proliferation and differentiation during erythropoiesis EMBO J 2006 25 2 357 366 10.1038/sj.emboj.7600934 16407974 PMC1383517

[B6] McGhee L Bryan J Elliott L Grimes HL Kazanjian A Davis JN Meyers S Gfi-1 attaches to the nuclear matrix, associates with ETO (MTG8) and histone deacetylase proteins, and represses transcription using a TSA-sensitive mechanism J Cell Biochem 2003 89 5 1005 1018 10.1002/jcb.10548 12874834

[B7] Melnick AM Westendorf JJ Polinger A Carlile GW Arai S Ball HJ Lutterbach B Hiebert SW Licht JD The ETO protein disrupted in t(8;21)-associated acute myeloid leukemia is a corepressor for the promyelocytic leukemia zinc finger protein Mol Cell Biol 2000 20 6 2075 2086 10.1128/MCB.20.6.2075-2086.2000 10688654 PMC110824

[B8] Schuh AH Tipping AJ Clark AJ Hamlett I Guyot B Iborra FJ Rodriguez P Strouboulis J Enver T Vyas P ETO-2 associates with SCL in erythroid cells and megakaryocytes and provides repressor functions in erythropoiesis Mol Cell Biol 2005 25 23 10235 10250 10.1128/MCB.25.23.10235-10250.2005 16287841 PMC1291220

[B9] Zhang J Kalkum M Yamamura S Chait BT Roeder RG E protein silencing by the leukemogenic AML1-ETO fusion protein Science 2004 305 5688 1286 1289 10.1126/science.1097937 15333839

[B10] Cai Y Xu Z Xie J Ham AJ Koury MJ Hiebert SW Brandt SJ Eto2/MTG16 and MTGR1 are heteromeric corepressors of the TAL1/SCL transcription factor in murine erythroid progenitors Biochem Biophys Res Commun 2009 390 2 295 301 10.1016/j.bbrc.2009.09.111 19799863 PMC2774815

[B11] Wang J Hoshino T Redner RL Kajigaya S Liu JM ETO, fusion partner in t(8;21) acute myeloid leukemia, represses transcription by interaction with the human N-CoR/mSin3/HDAC1 complex Proc Natl Acad Sci USA 1998 95 18 10860 10865 10.1073/pnas.95.18.10860 9724795 PMC27986

[B12] Hildebrand D Tiefenbach J Heinzel T Grez M Maurer AB Multiple regions of ETO cooperate in transcriptional repression J Biol Chem 2001 276 13 9889 9895 10.1074/jbc.M010582200 11150306

[B13] Gamou T Kitamura E Hosoda F Shimizu K Shinohara K Hayashi Y Nagase T Yokoyama Y Ohki M The partner gene of AML1 in t(16;21) myeloid malignancies is a novel member of the MTG8(ETO) family Blood 1998 91 11 4028 4037 9596646

[B14] Erickson P Gao J Chang KS Look T Whisenant E Raimondi S Lasher R Trujillo J Rowley J Drabkin H Identification of breakpoints in t(8;21) acute myelogenous leukemia and isolation of a fusion transcript, AML1/ETO, with similarity to Drosophila segmentation gene, runt Blood 1992 80 7 1825 1831 1391946

[B15] Miyoshi H Kozu T Shimizu K Enomoto K Maseki N Kaneko Y Kamada N Ohki M The t(8;21) translocation in acute myeloid leukemia results in production of an AML1-MTG8 fusion transcript EMBO J 1993 12 7 2715 2721 8334990 10.1002/j.1460-2075.1993.tb05933.x PMC413521

[B16] Guastadisegni MC Lonoce A Impera L Di Terlizzi F Fugazza G Aliano S Grasso R Cluzeau T Raynaud S Rocchi M CBFA2T2 and C20orf112: two novel fusion partners of RUNX1 in acute myeloid leukemia Leukemia 2010 24 8 1516 1519 10.1038/leu.2010.106 20520637

[B17] Nimer SD Moore MA Effects of the leukemia-associated AML1-ETO protein on hematopoietic stem and progenitor cells Oncogene 2004 23 24 4249 4254 10.1038/sj.onc.1207673 15156180

[B18] Lindberg SR Olsson A Persson AM Olsson I The Leukemia-associated ETO homologues are differently expressed during hematopoietic differentiation Exp Hematol 2005 33 2 189 198 10.1016/j.exphem.2004.10.011 15676213

[B19] Okumura AJ Peterson LF Lo MC Zhang DE Expression of AML/Runx and ETO/MTG family members during hematopoietic differentiation of embryonic stem cells Exp Hematol 2007 35 6 978 988 10.1016/j.exphem.2007.03.002 17533052

[B20] Meier N Krpic S Rodriguez P Strouboulis J Monti M Krijgsveld J Gering M Patient R Hostert A Grosveld F Novel binding partners of Ldb1 are required for haematopoietic development Development 2006 133 24 4913 4923 10.1242/dev.02656 17108004

[B21] Soler E Andrieu-Soler C de Boer E Bryne JC Thongjuea S Stadhouders R Palstra RJ Stevens M Kockx C van Ijcken W The genome-wide dynamics of the binding of Ldb1 complexes during erythroid differentiation Genes Dev 2010 24 3 277 289 10.1101/gad.551810 20123907 PMC2811829

[B22] Hamlett I Draper J Strouboulis J Iborra F Porcher C Vyas P Characterization of megakaryocyte GATA1-interacting proteins: the corepressor ETO2 and GATA1 interact to regulate terminal megakaryocyte maturation Blood 2008 112 7 2738 2749 10.1182/blood-2008-03-146605 18625887 PMC2556610

[B23] Chyla BJ Moreno-Miralles I Steapleton MA Thompson MA Bhaskara S Engel M Hiebert SW Deletion of Mtg16, a target of t(16;21), alters hematopoietic progenitor cell proliferation and lineage allocation Mol Cell Biol 2008 28 20 6234 6247 10.1128/MCB.00404-08 18710942 PMC2577421

[B24] Amann JM Chyla BJ Ellis TC Martinez A Moore AC Franklin JL McGhee L Meyers S Ohm JE Luce KS Mtgr1 is a transcriptional corepressor that is required for maintenance of the secretory cell lineage in the small intestine Mol Cell Biol 2005 25 21 9576 9585 10.1128/MCB.25.21.9576-9585.2005 16227606 PMC1265807

[B25] Ajore R Dhanda RS Gullberg U Olsson I The leukemia associated ETO nuclear repressor gene is regulated by the GATA-1 transcription factor in erythroid/megakaryocytic cells BMC Mol Biol 2010 11 38 10.1186/1471-2199-11-38 20487545 PMC2882371

[B26] Shivdasani RA Fujiwara Y McDevitt MA Orkin SH A lineage-selective knockout establishes the critical role of transcription factor GATA-1 in megakaryocyte growth and platelet development EMBO J 1997 16 13 3965 3973 10.1093/emboj/16.13.3965 9233806 PMC1170020

[B27] Vyas P Ault K Jackson CW Orkin SH Shivdasani RA Consequences of GATA-1 deficiency in megakaryocytes and platelets Blood 1999 93 9 2867 2875 10216081

[B28] Weiss MJ Keller G Orkin SH Novel insights into erythroid development revealed through in vitro differentiation of GATA-1 embryonic stem cells Genes Dev 1994 8 10 1184 1197 10.1101/gad.8.10.1184 7926723

[B29] Elagib KE Xiao M Hussaini IM Delehanty LL Palmer LA Racke FK Birrer MJ Shanmugasundaram G McDevitt MA Goldfarb AN Jun blockade of erythropoiesis: role for repression of GATA-1 by HERP2 Mol Cell Biol 2004 24 17 7779 7794 10.1128/MCB.24.17.7779-7794.2004 15314183 PMC506977

[B30] Lutterbach B Sun D Schuetz J Hiebert SW The MYND motif is required for repression of basal transcription from the multidrug resistance 1 promoter by the t(8;21) fusion protein Mol Cell Biol 1998 18 6 3604 3611 9584201 10.1128/mcb.18.6.3604 PMC108942

[B31] Erickson PF Robinson M Owens G Drabkin HA The ETO portion of acute myeloid leukemia t(8;21) fusion transcript encodes a highly evolutionarily conserved, putative transcription factor Cancer Res 1994 54 7 1782 1786 8137293

[B32] Lederfein D Yaffe D Nudel U A housekeeping type promoter, located in the 3 region of the Duchenne muscular dystrophy gene, controls the expression of Dp71, a major product of the gene Hum Mol Genet 1993 2 11 1883 1888 10.1093/hmg/2.11.1883 8281151

[B33] Fraizer GC Wu YJ Hewitt SM Maity T Ton CC Huff V Saunders GF Transcriptional regulation of the human Wilms' tumor gene (WT1). Cell type-specific enhancer and promiscuous promoter J Biol Chem 1994 269 12 8892 8900 8132626

[B34] Pinte S Guerardel C Deltour-Balerdi S Godwin AK Leprince D Identification of a second G-C-rich promoter conserved in the human, murine and rat tumor suppressor genes HIC1 Oncogene 2004 23 22 4023 4031 10.1038/sj.onc.1207504 15007385

[B35] Zhao C He X Tian C Meng A Two GC-rich boxes in huC promoter play distinct roles in controlling its neuronal specific expression in zebrafish embryos Biochem Biophys Res Commun 2006 342 1 214 220 10.1016/j.bbrc.2006.01.134 16472769

[B36] Dynan WS Sazer S Tjian R Schimke RT Transcription factor Sp1 recognizes a DNA sequence in the mouse dihydrofolate reductase promoter Nature 1986 319 6050 246 248 10.1038/319246a0 3945313

[B37] Martin DI Zon LI Mutter G Orkin SH Expression of an erythroid transcription factor in megakaryocytic and mast cell lineages Nature 1990 344 6265 444 447 10.1038/344444a0 2320112

[B38] Shimizu R Yamamoto M Gene expression regulation and domain function of hematopoietic GATA factors Semin Cell Dev Biol 2005 16 1 129 136 10.1016/j.semcdb.2004.11.001 15659347

[B39] Leonard M Brice M Engel JD Papayannopoulou T Dynamics of GATA transcription factor expression during erythroid differentiation Blood 1993 82 4 1071 1079 8353273

[B40] Fujiwara T O'Geen H Keles S Blahnik K Linnemann AK Kang YA Choi K Farnham PJ Bresnick EH Discovering hematopoietic mechanisms through genome-wide analysis of GATA factor chromatin occupancy Mol Cell 2009 36 4 667 681 10.1016/j.molcel.2009.11.001 19941826 PMC2784893

[B41] Tsai FY Keller G Kuo FC Weiss M Chen J Rosenblatt M Alt FW Orkin SH An early haematopoietic defect in mice lacking the transcription factor GATA-2 Nature 1994 371 6494 221 226 10.1038/371221a0 8078582

[B42] Simon MC Pevny L Wiles MV Keller G Costantini F Orkin SH Rescue of erythroid development in gene targeted GATA-1- mouse embryonic stem cells Nat Genet 1992 1 2 92 98 10.1038/ng0592-92 1302015

[B43] Yu C Cantor AB Yang H Browne C Wells RA Fujiwara Y Orkin SH Targeted deletion of a high-affinity GATA-binding site in the GATA-1 promoter leads to selective loss of the eosinophil lineage in vivo J Exp Med 2002 195 11 1387 1395 10.1084/jem.20020656 12045237 PMC2193547

[B44] Grass JA Boyer ME Pal S Wu J Weiss MJ Bresnick EH GATA-1-dependent transcriptional repression of GATA-2 via disruption of positive autoregulation and domain-wide chromatin remodeling Proc Natl Acad Sci USA 2003 100 15 8811 8816 10.1073/pnas.1432147100 12857954 PMC166395

[B45] Pal S Cantor AB Johnson KD Moran TB Boyer ME Orkin SH Bresnick EH Coregulator-dependent facilitation of chromatin occupancy by GATA-1 Proc Natl Acad Sci USA 2004 101 4 980 985 10.1073/pnas.0307612100 14715908 PMC327128

[B46] Gardini A Cesaroni M Luzi L Okumura AJ Biggs JR Minardi SP Venturini E Zhang DE Pelicci PG Alcalay M AML1/ETO oncoprotein is directed to AML1 binding regions and co-localizes with AML1 and HEB on its targets PLoS Genet 2008 4 11 e1000275 10.1371/journal.pgen.1000275 19043539 PMC2577924

[B47] Choi Y Elagib KE Delehanty LL Goldfarb AN Erythroid inhibition by the leukemic fusion AML1-ETO is associated with impaired acetylation of the major erythroid transcription factor GATA-1 Cancer Res 2006 66 6 2990 2996 10.1158/0008-5472.CAN-05-2944 16540647

[B48] Tonks A Pearn L Tonks AJ Pearce L Hoy T Phillips S Fisher J Downing JR Burnett AK Darley RL The AML1-ETO fusion gene promotes extensive self-renewal of human primary erythroid cells Blood 2003 101 2 624 632 10.1182/blood-2002-06-1732 12393523

[B49] Lennartsson A Pieters K Ullmark T Vidovic K Gullberg U AML-1, PU.1, and Sp3 regulate expression of human bactericidal/permeability-increasing protein Biochem Biophys Res Commun 2003 311 4 853 863 10.1016/j.bbrc.2003.10.067 14623259

[B50] Andrews NC Faller DV A rapid micropreparation technique for extraction of DNA-binding proteins from limiting numbers of mammalian cells Nucleic Acids Res 1991 19 9 2499 10.1093/nar/19.9.2499 2041787 PMC329467

[B51] Ginzinger DG Gene quantification using real-time quantitative PCR: an emerging technology hits the mainstream Exp Hematol 2002 30 6 503 512 10.1016/S0301-472X(02)00806-8 12063017

[B52] Dhanda RS Lindberg SR Olsson I The human SIN3B corepressor forms a nucleolar complex with leukemia-associated ETO homologues BMC Mol Biol 2008 9 8 10.1186/1471-2199-9-8 18205948 PMC2266940

